# Supplementary material for: Single-centre, single-blinded, randomised, parallel group, feasibility study protocol investigating if mandibular advancement device treatment for obstructive sleep apnoea can reduce nocturnal gastro-oesophageal reflux (MAD-Reflux trial)
Source: BMJ Open. 2023 Aug 24;13(8):e076661. doi: 10.1136/bmjopen-2023-076661 (PMC10450077; doi:10.1136/bmjopen-2023-076661)
Supplement: Supplementary data [file bmjopen-2023-076661supp001.pdf]

		Pre-Screening Visit	Visit 1: Screening visit		Visit 2 Intervention			Visit 3 or more	Final assessment visit	
					CPAP	MAD				
			Day 1	Day 2		App 1	App 2		Day 1	Day 2
**ENROLMENT**	Eligibility screen	x								
	Patient information sheet given and consent for additional screening procedures	x								
	RDQ and dental screen	x								
	Advice re reflux meds and recording diet prior to screening/final appointment	x						x		
	Full written informed consent		x							
	Dental examination, manometry and placement of pH catheter		x						x	
	Receive Watch-PAT home sleep monitoring		x						x	
	Return sleep monitoring device and remove pH catheter			x						x
	24h food intake recording while pH probe is placed		x	x					x	x
	Eligibility confirmed and randomisation			x						

INTERVENTIONS	CPAP									
	CPAP mask fitted and instructions given				x					
	MAD									
	Digital oral impressions taken for MAD and delivered 3 weeks later					x	x			
	Device titration appointments							x		
ASSESSMENTS	Documenting numbers for feasibility outcomes on those approached, screened and completed the trial	x		x						x
	Qualitative Interviews about participant views in taking part in the study	x		x						x
	RSI, ESS, Leicester Cough Questionnaire, QoL questionnaires		x						x	
	Change in percentage acid contact time pH < 4 with the device in situ									x
	Hours that device is worn during sleep									x

Table 1. Schedule of events, interventions and assessments for the MAD Reflux Trial according to Spirit Guidelines
