# Supplementary material for: Single-centre, single-blinded, randomised, parallel group, feasibility study protocol investigating if mandibular advancement device treatment for obstructive sleep apnoea can reduce nocturnal gastro-oesophageal reflux (MAD-Reflux trial)
Source: BMJ Open. 2023 Aug 24;13(8):e076661. doi: 10.1136/bmjopen-2023-076661 (PMC10450077; doi:10.1136/bmjopen-2023-076661)
Supplement: Author's manuscript [file bmjopen-2023-076661.draft_revisions.pdf]

BMJ Open

BMJ Open is committed to open peer review. As part of this commitment we make the peer review history of every article we publish publicly available.

When an article is published we post the peer reviewers' comments and the authors' responses online. We also post the versions of the paper that were used during peer review. These are the versions that the peer review comments apply to.

The versions of the paper that follow are the versions that were submitted during the peer review process. They are not the versions of record or the final published versions. They should not be cited or distributed as the published version of this manuscript.

BMJ Open is an open access journal and the full, final, typeset and author-corrected version of record of the manuscript is available on our site with no access controls, subscription charges or pay-per-view fees (http://bmjopen.bmj.com).

If you have any questions on BMJ Open's open peer review process please email info.bmjopen@bmj.com

BMJ Open

Can Mandibular Advancement Devices Reduce Nocturnal Reflux: A Feasibility Study Protocol.

Journal:	*BMJ Open*
Manuscript ID	bmjopen-2023-076661
Article Type:	Protocol
Date Submitted by the Author:	13-Jun-2023
Complete List of Authors:	O'Toole, Saoirse; King's College London, ; University College Dublin,<br>Moazzez, Rebecca; University of the Pacific Arthur A Dugoni School of Dentistry<br>Wojewodka, Gabriella; King's College London, Oral and Clinical Research Unit<br>Zeki, Sebastian; Guy's and St Thomas' Hospitals NHS Trust<br>Jafari, Jafar; Guy's and St Thomas' Hospitals NHS Trust<br>Hope, Katherine; Hope2Sleep<br>Brand, Andrew; Bangor University, Statistics<br>Hoare, Zoe; Bangor University, North Wales Organisation for Randomised Trials in Health<br>Scott, Suzanne; Queen Mary University of London Wolfson Institute of Population Health<br>Doungsong, Kodchawan; Bangor University, Centre for Health Economics and Medicines Evaluation<br>Ezeofar, Victory; Bangor University, Centre for Health Economics and Medicines Evaluation<br>Edwards, Rhiannon; Bangor University, Centre for Health Economics & Medicines Evaluation<br>Drakatos, Panagis; Guy's and St Thomas' Hospitals NHS Trust<br>Steier, Joerg; Guy`s & St Thomas` NHS Foundation Trust, Lane Fox Unit / Sleep Disorders Centre; King`s College London, School of Medicine
Keywords:	Health economics < HEALTH SERVICES ADMINISTRATION & MANAGEMENT, SLEEP MEDICINE, GASTROENTEROLOGY, Feasibility Studies, ORAL MEDICINE

SCHOLARONE™
Manuscripts

Can Mandibular Advancement Devices Reduce Nocturnal Reflux: A Feasibility Study Protocol.

Abstract

Introduction: Just under half of patients with obstructive sleep apnoea (OSA) also have gastro-oesophageal reflux disease (GORD). These conditions appear to be inter-related and Continual Positive Airway Pressure (CPAP) therapy, the gold standard treatment for OSA to prevent airway collapse, has been shown to reduce GORD. The impact of Mandibular Advancement Devices (MADs), a second line therapy for OSA, on GORD has yet to be investigated and therefore a feasibility study is needed.

Methods: This will be a single-centre, single-blinded, tertiary-care based, interdisciplinary, parallel randomised controlled study. Potential OSA participants presenting to the sleep department will be pre-screened for GORD using validated questionnaires, consented and invited to receive simultaneous home sleep and oesophageal pH monitoring. Those with confirmed OSA and GORD (n=44) will be randomly allocated to receive either CPAP (n=22) or MAD therapy (n=22). Following successful titration and 3 weeks customisation period, participants will repeat the simultaneous sleep and oesophageal pH monitoring while wearing the device. The number of patients screened for recruitment, drop-out rates, patient feedback of the study protocol, costs of interventions and clinical information to inform a definitive study design, will be investigated.

Ethics and Dissemination: Health Research Authority approval has been obtained from the Nottingham 2 Research Ethics Committee, ref:22/EM/0157 and the trial has been registered on ISRCTN (https://doi.org/10.1186/ISRCTN16013232). Definitive findings about the feasibility of doing 24 hr pH oesophageal monitoring whilst doing a home sleep study will be disseminated via clinical and research networks facilitating valuable insights into the simultaneous management of both conditions.

Keywords: Obstructive sleep apnoea, gastro-oesophageal reflux, mandibular advancement devices, health economics, economic evaluation, process evaluation, feasibility study

Strengths and Limitations:

- This is a well-designed, interdisciplinary protocol with potential to provide information on treatment options for two highly prevalent conditions: Gastro-oesophageal reflux and Obstructive Sleep Apnoea

For peer review only

- Health economics will also be assessed.
- Due to uncertainties regarding patient recruitment and clinical effect sizes a feasibility study is required prior to a definitive trial.

Introduction

One billion people worldwide and 8 million people in the UK (24.5% of the population) are estimated to have Obstructive Sleep Apnoea (OSA)[1]. It is the most common sleep disorder, characterised by frequent pauses in breathing (apnoeas and hypopnoeas) during sleep due to airway blockage caused by intermittent relaxation of the throat muscles. Sleep becomes broken, so in the daytime patients complain of sleepiness, fatigue, and have other symptoms such as difficulty concentrating, memory impairment, feeling irritable and depressed [2]. Their own and their bed partner's quality of life is reduced [2]. Patients are at a higher risk of type 2 diabetes, high blood pressure, heart disease, strokes, and dying of all causes compared to people without OSA. This risk increases with OSA severity; with severe OSA, the risk of cardiac events and strokes is doubled and of cardiac death is tripled [3]. People with untreated OSA also put the public in danger as they have more road traffic accidents than people without OSA and compared to themselves once on treatment [4,5]. The societal level-costs of undiagnosed OSA in the United States alone amounted to over $150 billion per year [6]. In Italy, OSA causes an economic burden ranging from €10.7 to €32 billion per year [7].

Gastro-Oesophageal Reflux Disease (GORD), defined by the Montreal consensus as "a condition that develops when the reflux of stomach contents causes troublesome symptoms and/or complications" with oesophageal/typical and extraoesophageal/ atypical symptoms [8] is also common in the UK. There is a strong association between obstructive sleep apnoea and GORD, partly due to common risk factors such as obesity, high alcohol consumption and diet but also due to the impact that apnoeic events have on the lower oesophageal sphincter [9]. A recent meta-analysis estimated that 45.2% of OSA patients had episodes of extra-oesophageal reflux independent of BMI [9]. Around 40-81% of patients suffering from GORD report that their symptoms occur during sleep [10]. There is a growing body of evidence that Continuous Positive Airway Pressure (CPAP) therapy, the gold standard therapy for OSA [11], can reduce levels of gastro-oesophageal disease [12–15] by maintaining a patent airway, thus reducing intrathoracic

For peer review only - http://bmjopen.bmj.com/site/about/guidelines.xhtml

pressure differentials. CPAP therapy increases the baseline lower oesophageal sphincter barrier pressure during sphincter relaxation and decreases the duration of sphincter relaxation [16]. Mandibular advancement devices (MADs), a second line therapy, also maintain a patent airway which may also have a similar impact on intrathoracic pressure differentials and duration of lower oesophageal sphincter relaxation. In addition, the greater compliance observed with MADs may mean that reflux is suppressed for a greater proportion of the night. However, both theories remain untested to date. We do not have sufficient data to gauge an effect size for a definitive powered trial. We do not know if patients are willing to wear multiple devices and tests whilst trying to sleep and whether acceptability will influence the data. We are also unaware of the most appropriate quality of life measure to accurately assess patient impact and health economics. Therefore, a feasibility trial is needed prior to a definitive evaluation.

From a cost perspective, CPAP and MADs are both cost-effective treatments[17]. CPAP therapy has been shown to generate an incremental cost-effectiveness ratio (ICER) below £5,000 per quality-adjusted life-year (QALY) gained [18]. Using CPAP over a period of 14 years resulted in saving National Health Service (NHS) costs (nearly £1,000 per patient) and generated health benefits for people with OSA e.g. reduction in stroke risk and road traffic accidents[19].

In the UK, the yearly medication costs of GORD treatment are around £200 million for histamine receptor antagonists (H_2RAs) and £300 million for proton pump inhibitors (PPIs)[20 22]. The cost implication of the impact of CPAP therapy or MAD therapy on GORD has yet to be evaluated.

The overall aim of this project is to conduct a feasibility study to assess patient recruitment and tolerance of the trial. The secondary aims are to calculate an estimate of the primary outcome effect to determine the sample size needed for the definitive trial if the progression criteria are met, to determine the optimum measure of quality-of-life impact for this patient cohort.

Methods and Analysis

Study Design

This will be a single-centre, single-blinded, tertiary care based, interdisciplinary parallel 1:1 randomised controlled study. Health Research Authority and Ethics approval has been obtained from the Nottingham 2 Research Ethics Committee, ref: 22/EM/0157 and the trial has been registered on ISRCTN (reference 16013232, https://doi.org/10.1186/ISRCTN16013232).

Setting

For peer review only

Participants will be recruited from King’s Health Partners sleep services at Guy’s and St Thomas’s Foundation Trust (GSTT, London, UK).

Outcomes:

Primary outcome measures

1. Percentage of approached patients who were screened for the trial measured using the percentage of patients eligible of those screened at Day 2 of the screening visit.

2. Percentage of eligible patients who were randomised measured using the percentage of screened patients who met the eligibility criteria versus those who were randomised at the randomisation timepoint.

3. Percentage of patients who completed the trial measured using number of patients who were randomised versus those who completed the trial at Day 2 of the final visit.

Primary clinical outcome to signal efficacy.

1. Change in percentage acid contact time pH < 4 with the device in situ measured using nocturnal data from 24-hour pH monitoring at initial gastroenterology screening visit and follow-up gastroenterology assessment post therapy.

Secondary outcome measures

1. Patient acceptability of the trial measured using qualitative interviews throughout the trial.

2. Hours that device is worn during sleep measured using the average number of hours worn per night as detected from the output from the therapeutic device throughout the trial period.

3. To assess the most sensitive quality of life questionnaire prior to the full trial by recording EQ-5D-5L, ICE-CAP A, and SWEMWBS at baseline and post intervention.

4. Rating of both therapies measured using a Visual Analog Scale (VAS) at Day 2 of final assessment visit (trial completion).

5. Number of potential participants identified by the care team with and without screening of referral letters measured using number of participants identified on clinics before the pre-screening clinic and after the pre-screening.

6. Change in Reflux Symptom Index (RSI) using changes detected in the RSI validated questionnaire at baseline and post intervention.

7. Change in Epworth Sleepiness Scale (ESS) using changes detected in the ESS validated questionnaire at baseline and post intervention.

8. Change in cough using changes detected in the validated Leicester Cough Questionnaire at baseline and post intervention.

For peer review only

9. To identify and measure indicative costs and outcomes and select suitable economic outcomes measured by documenting health resource use including intervention costs, appointment times and attending health care practitioners throughout the trial.

10. To determine the acceptability of the intervention and economic data collection methods measured using qualitative questions at the qualitative interview (various timepoints).Primary outcomes

Participants and recruitment

Potential participants will be identified by the direct care team either by running a search text function to screen their referral letters or by their consultation in clinic. Patients referred for investigation of OSA, who also have a previously confirmed diagnosis of GORD with a 24 hour pH study or typical heartburn and/ or regurgitation >3 times per week and Reflux Disease Questionnaire (RDQ) questionnaire score of >50%, will be informed by their direct care team that they may be eligible to be included in the study and asked if they would like to speak to a research dentist or nurse about participation. The pre-screening questions for dental examination will ask patients if they have at least 10 teeth in each jaw, no loose teeth, fillings or caps or obvious holes in your teeth. Interested participants who meet the pre-screening criteria, will be provided with a patient information sheet of the entire trial and invited to have a dental screening during their gastroenterology appointment, followed by a combined home sleep study and 24-hour pH impedance oesophageal monitoring. It will be explained that participation in the trial will be dependent upon meeting the strict inclusion criteria but they will be reimbursed for the screening appointment.

Inclusion/Exclusion Criteria

Patients will be included in the study if they are:

1. Aged 18 or over,
2. Confirmed OSA with Apnoea-Hypopnoea Index (AHI) score between 10 and 50
3. Confirmed gastro-oesophageal reflux disease with greater than 6 percent of acid exposure time <pH 4 over 24 hours,
4. No previous CPAP or MAD therapy,
5. Sufficient healthy teeth to support a mandibular advancement device i.e. 10 teeth in each jaw, no periodontal pockets >5, no frank cavitation or loose crowns/bridges,
6. Willing and able to provide informed consent to the study.

Patients will be ineligible if:

For peer review only

1. Pregnant or breast feeding,
2. Unable or unwilling to stop GORD medication 2 days prior to assessment or unable to undergo manometry and pH impedance testing,
3. Known liver disease or oesophageal/gastric varices,
4. Previous surgery or intervention for reflux such as fundoplication which may preclude pH impedance testing,
5. Any previous treatment for oesophageal neoplasia,
6. Unable/unwilling to tolerate either a CPAP mask or a mandibular advancement device,
7. Medical history likely to impact on 24-hour impedance testing e.g bulimia nervosa,
8. Participation in other research within previous 30 days.

Study screening procedures

Following written informed consent, participants will undergo assessments at a screening visit to ensure eligibility is met. Participants will be informed that proton pump inhibitors will need to be stopped 7 days prior and H2-receptor antagonists or antacids 48 hours prior to this visit. Participants will complete a reflux symptom index questionnaire, Epworth Sleepiness Scale questionnaire, Leicester Cough Questionnaire and several QoL questionnaires. A full dental exam will be conducted to ensure that participants are dentally fit to wear a MAD if allocated to this intervention. For the gastroenterology assessment, the patient will initially need to undergo high resolution manometry (HRM). Following local analgesia of the nares the catheter will be introduced trans-nasally and the patient instructed to drink water through a straw whilst the HRM catheter is advanced to the stomach. The HRM catheter depth will be adjusted to ensure manometric visual of the upper oesophageal sphincter (UOS), the gastro-oesophageal junction (GOJ) and gastric pressures. 10 single swallows of 5ml will be performed with each being 20 seconds apart. Each 5ml water swallow will be assessed in accordance with Chicago classification (version 3) using Manoview software (version 3) (Sierra Scientific Instruments). The HRM catheter will then be removed.

Patients will then undergo reflux monitoring using Sandhill Scientific multichannel impedance pH catheters (ZANBG-44) which are inserted trans-nasally after applying local anaesthesia (xylocaine). The dual pH sensors of the catheter will be positioned 5cm below and above the manometric lower oesophageal sphincter (LOS). The impedance sensors will be positioned above the LOS by 3cm, 5cm, 9cm, 15cm and 19cm. The data will be captured by ZepHrTM recording device.

For peer review only

For the 24 hours while the probe is inserted, the participant will be asked to complete a food diary as food and drink will impact on their reflux. Stopping reflux medication and the food diary are part of standard care for this procedure.

Following placement of the catheter, participants will be invited for their home sleep study. A type 3 sleep study device, the WatchPAT 200 (WP200; Itamar Medical Ltd., Caesarea, Israel) will be given in addition to comprehensive instructions on how to perform a home sleep study. A 24h number for technical support will be provided to the patient. After their overnight sleep study, they will return the following day to the gastroenterology department for removal of the probe and return of the WatchPAT.

The gastroenterology data will be captured by ZepHrTM recording device and data will be analysed using the BioVIEW Analysis software (5.7.1.0). The sleep study data will be analysed by a qualified sleep technician.

Randomisation Procedures

Participants who meet the inclusion/exclusion criteria will be allocated 1:1 using fixed-block randomisation in the King's Clinical Trials Unit (KCTU) to either the CPAP (n = 22) or the MAD (n = 22) arm.

Interventions

The intervention will consist of either a MAD (n=22, SomnoMed Avant, Somnomed UK) with the dental sleep medicine department or CPAP (n=22, S8/S9, ResMed Ltd, Sydney, Australia) with the sleep medicine department.

Titration of Devices and Repeat Assessment

Some adjustment of the devices may be needed to ensure comfort and efficacy of the device. This will be done as routine care and scheduled as needed.

Following successful titration for each device and a three-week customisation period, the 24-hour impedance monitoring and home sleep study will be repeated. As at visit 1, participants will be asked to cease their proton pump inhibitors, H2-receptor antagonists, or antacids. A dental examination will be carried out by the research dentist to ensure there have been no changes in the oral cavity. Participants will be asked to review their diet diary from the day they first did the test and repeat what they ate or drank as closely as possible for the day. That afternoon the participant will attend the oesophageal physiology laboratory. Participants will repeat the reflux

For peer review only

symptom index questionnaire, Epworth Sleepiness Scale questionnaire, Leicester Cough Questionnaire and a QoL questionnaire. The pH impedance testing and Watch-PAT testing will be repeated as described above. Compliance levels for the MAD and CPAP therapy on the same night will be obtained.

Upon returning the next day to have their pH probe removed and to return the Watch-PAT, participants will be asked to complete a questionnaire about their views in taking part in the study.

Blinding

This is a single-blinded (statistician) study. The patient and clinician cannot be blinded to the group allocation due to the nature of the therapeutic devices used. However, the objective, computer-generated clinical measurement outcome in for the definitive trial is less likely to be subject to bias.

Data collection and management

Clinical, demographic and questionnaire data will be collected on case report forms. Clinical data from manometry, impedance testing and sleep study will be obtained through patient electronic records and recorded on case report forms. Paper forms will be used, and data will be recorded on electronic case report forms held in a database using MACRO 4.0 (Infermed) and maintained by the KCTU. Data checks will be performed by the trial manager and the trial statistician.

Qualitative Interviews

To enhance our understanding of acceptability of recruitment processes, screening procedures, and of taking part in the trial we will conduct telephone interviews with participants (approx. n=16) over the course of the trial. We will use a sampling matrix to include patients who elected not to take part, eligible patients who were screened but did not take part in the trial, patients who could not tolerate the device or discontinued the trial for other reasons, and those who completed the trial.

Using the Theoretical Framework of Acceptability [21] to underpin the interview guide, participants will be asked about different domains of acceptability (e.g. affective attitude, burden, coherence, perceived effectiveness) for each element of the trial, including recruitment, randomisation, appointments and procedures, use of devices. Participants will also be asked about barriers to trial participation, potential improvements, and how to create advocacy for the trial amongst stakeholders. Participants will receive an additional £25 if they take part in this 25-minute

For peer review only

interview. The interviews will be audio-recorded, anonymised and professionally transcribed verbatim, in preparation for Framework Analysis [22] whereby a thematic framework is developed and applied to the transcripts, allowing systematic analysis and interpretation of the qualitative data. Data will be managed in NVivo.

Health Economic Measures

During the feasibility phase, the best way of collecting relevant service costs, and patient borne costs, from both an NHS and a wider societal perspective will be investigated to inform a future full trial context [23–25]. We will consider to what extent this intervention constitutes a complex intervention, drawing on guidance from the MRC for the guidance for complex interventions [26]. In the context of the patient, we will pilot the EQ-5D (5L) [27], ICECAP-A [28,29] and the Short Warwick–Edinburgh Mental Wellbeing Scale, SWEMWBS [30] which could be used in a full RCT to calculate cost per Quality Adjusted Life Years (QALYs) and wider outcomes. We will determine if these are sufficiently sensitive to measure change in the patient group. We will also determine the appropriate deterministic sensitivity analysis in a full economic analysis in this patient group. We will pay attention to distributive cost-effectiveness analysis opportunities in a full trial [31].

Our base-case analysis will explore the likely total costs (intervention costs and medical costs) and outcomes (health resource service use and health-related quality of life) of using MADs compared to CPAP therapy in people with OSA who have nocturnal GORD in a future full trial. We will also explore if these outcome measures accurately reflect the conditions of the patients.

We will write a report to the funder and papers for peer-review publication following the Consolidated Health Economic Evaluation Reporting Standards (CHEERS) statement [32] and the Assessment of the Validation Status of Health-Economic Decision Model (AdVISHE) [33]. The health economic feasibility study will be reported in accordance with the Consolidated Standards of Reporting Trials (CONSORT) extension [34].

A full schedule of events is given in Table 1 and the patient flow diagram with feasibility outcomes is illustrated in Figure 1.

Adverse events

All adverse events (AEs) will be recorded from the time of randomisation. AEs will be classified according to severity and whether related to the study intervention. All Serious Adverse Events SAEs will be reported immediately by the Chief Investigator and, no later than 24hrs, to the R&D office (Sponsor) and main REC.

For peer review only

Sample Size

There are several studies investigating change in % acid exposure from baseline whilst wearing the CPAP device. Samples sizes suggested from current data based on change in total acid were between 86 to 168 per group (excluding any attrition)[12,35]. Using the smallest effect size published (Tawk et al.[12]), assuming a one-sided non-inferiority design with a margin of 4.15 (% total acid) together with a SD of 10.8 with a power of 90% and alpha of 2.5% for a definitive study, this would require a sample of 288 (without attrition). Considering the likelihood of the main study finding this an effect of this size within an 80% confidence interval, then an approach taken by Cocks and Torgerson [36] requires a feasibility sample equivalent to approximately 9% of the proposed definitive sample requiring 26 participants. Accommodating an overall attrition rate, including tolerance to the devices, of 40% requires a sample of 44 to be randomised. A sample of 44 will also give us a 95% confidence interval of +/- 14% around the attrition rate.

Statistical Analysis

Baseline characteristics will be summarized for all participants within the trial arms.

The feasibility outcomes include:

1. Percentage of screened patients who were eligible for the trial.
2. Percentage of eligible patients who agreed to participate.
3. Percentage of patients who completed the trial.

We will then assess one clinical outcome to assess signal of efficacy which is the change in percentage acid contact time pH < 4 with the device in situ. Participants' uptake of and adherence to both CPAP and MAD, as well as follow-up rates, will be summarized and presented as percentages. Although determining differences in clinical outcomes between the arms is not the primary purpose of this feasibility study, comparisons will be undertaken to investigate the feasibility of studying these outcomes and to calculate potential estimates and 95% confidence intervals. As recommended in guidelines for good practice for the analysis of pilot studies [37], the focus of the results will be on the estimates of the treatments rather than statistical significance and as such no hypothesis testing will be undertaken. Differences between the two comparison groups will be presented in the form of an unadjusted mean difference for continuous outcomes, and an odds ratio for binary outcomes, with their associated 95% confidence intervals. These comparisons will be made on an intention to treat basis with consideration given to per protocol

For peer review only

analysis as sensitivity. While every effort will be made to minimise missing data, assessment of the levels of missing data will indicate suitability of measures to be continued into the definitive trial.

Discussion

Given the increasing prevalence of both OSA and GORD, it is important to further investigate the inter-relationship between the conditions and the impact of OSA treatment on GORD. While there is evidence that CPAP reduces GORD, the effect size is unclear, and the impact of MADs on GORD is entirely un-investigated.

This feasibility trial is designed as a first step to provide this important information for patients deciding on treatment choices and the prescribing sleep clinician. This trial will give us insights into the feasibility for testing patients, patient experience, the mechanism of action and the overall effect. We will also explore health economic measures to best capture the cost of services versus the value obtained for patients and health care providers.

Patient and public involvement statement

The trial was designed to generate evidence for patients with both OSA and GORD who are deciding on their treatment options. Our patient co-applicant is highly active within the OSA community with both feedback and dissemination means at a national level (Hope2Sleep Charity). We have an active PPI group who were also involved in the review and development of study protocol and patient information sheets. Patient advocacy is an important part of our oversight committee and our patient co-applicant is in charge of ensuring the patient perspective is presented throughout the duration of the trial lifecycle. All findings from the study will be made available for dissemination to the public.

Authors' Contributions

SOT, RM, ZS, JJ, GW, KH, AB, ZH, SS, RTE, VE, KD and JS contributed to the conception and design of the study. SOT drafted the manuscript. All authors critically revised the manuscript. All authors gave their final approval and agree to be accountable for all aspects of the work.

Conflicts of Interest

The authors have no conflicts of interest to declare

Funding Statement

For peer review only

This project is funded by the National Institute for Health and Care Research (NIHR) under its Research for Patient Benefit (RfPB) Programme (Grant Reference Number NIHR202744). The views expressed are those of the authors and not necessarily those of the NIHR or the Department of Health and Social Care.

Acknowledgement

Thanks to Dr Catherine Lawrence for reading support for RTE.

		Pre-Screening Visit	Visit 1: Screening visit		Visit 2 Intervention			Visit 3 or more	Final assessment visit	
					CPAP	MAD				
			Day 1	Day 2		App 1	App 2		Day 1	Day 2
**ENROLMENT**	Eligibility screen	x								
	Patient information sheet given and consent for additional screening procedures	x								
	RDQ and dental screen	x								
	Advice re reflux meds and recording diet prior to screening/final appointment	x						x		
	Full written informed consent		x							
	Dental examination, manometry and placement of pH catheter		x						x	
	Receive Watch-PAT home sleep monitoring		x						x	
	Return sleep monitoring device and remove pH catheter			x						x

For peer review only

	24h food intake recording while pH probe is placed		x	x					x	x
	Eligibility confirmed and randomisation			x						
**INTERVENTIONS**	CPAP<br>CPAP mask fitted and instructions given				x					
	MAD<br>Digital oral impressions taken for MAD and delivered 3 weeks later					x	x			
	Device titration appointments							x		
**ASSESSMENTS**	Documenting numbers for feasibility outcomes on those approached, screened and completed the trial	x		x						x
	Qualitative Interviews about participant views in taking part in the study	x		x						x
	RSI, ESS, Leicester Cough Questionnaire, QoL questionnaires		x						x	
	Change in percentage acid contact time pH < 4 with the device in situ									x
	Hours that device is worn during sleep									x

For peer review only

Table 1. Schedule of events for the MAD Reflux Trial

1. Wimms AJ, Kelly JL, Turnbull CD, et al. Continuous positive airway pressure versus standard care for the treatment of people with mild obstructive sleep apnoea (MERGE): a multicentre, randomised controlled trial. *Lancet Respir Med*. 2020;8(4):349-358. doi:10.1016/S2213-2600(19)30402-3
2. Moyer CA, Sonnad SS, Garetz SL, Helman JI, Chervin RD. Quality of life in obstructive sleep apnea: A systematic review of the literature. *Sleep Med*. 2001;2(6):477-491. doi:10.1016/S1389-9457(01)00072-7
3. Xie C, Zhu R, Tian Y, Wang K. Association of obstructive sleep apnoea with the risk of vascular outcomes and all-cause mortality: A meta-analysis. *BMJ Open*. 2017;7(12). doi:10.1136/bmjopen-2016-013983
4. Karimi M, Hedner J, Häbel H, Nerman O, Grote L. Sleep apnea related risk of motor vehicle accidents is reduced by continuous positive airway pressure: Swedish traffic accident registry data. *Sleep*. 2015;38(3):341-349. doi:10.5665/sleep.4486
5. Garbarino S, Durando P, Guglielmi O, et al. Sleep apnea, sleep debt and daytime sleepiness are independently associated with road accidents. A cross-sectional study on truck drivers. *PLoS One*. 2016;11(11). doi:10.1371/journal.pone.0166262
6. Natsky AN, Vakulin A, Coetzer CLC, McEvoy RD, Adams RJ, Kaambwa B. Economic evaluation of diagnostic sleep studies for obstructive sleep apnoea: a systematic review protocol. *Syst Rev*. 2021;10(1). doi:10.1186/s13643-021-01651-3
7. Borsoi L, Armeni P, Donin G, Costa F, Ferini-Strambi L. The invisible costs of obstructive sleep apnea (OSA): Systematic review and cost-of-illness analysis. *PLoS One*. 2022;17(5 May). doi:10.1371/journal.pone.0268677
8. Vakil N, van Zanten S v., Kahrilas P, et al. The Montreal definition and classification of gastroesophageal reflux disease: A global evidence-based consensus. *American Journal of Gastroenterology*. 2006;101(8):1900-1920. doi:10.1111/j.1572-0241.2006.00630.x
9. Magliulo G, Iannella G, Polimeni A, et al. Laryngopharyngeal reflux in obstructive sleep apnoea patients: Literature review and meta-analysis. *American Journal of Otolaryngology - Head and Neck Medicine and Surgery*. 2018;39(6):776-780. doi:10.1016/j.amjoto.2018.09.006
10. Orr WC. Review article: Sleep-related gastro-oesophageal reflux as a distinct clinical entity. *Aliment Pharmacol Ther*. 2010;31(1):47-56. doi:10.1111/j.1365-2036.2009.04124.x
11. NICE guideline [NG202]. *Obstructive Sleep Apnoea/ Hypopnoea Syndrome and Obesity Hypoventilation Syndrome in over 16s NICE Guideline*.; 2021. www.nice.org.uk/guidance/ng202
12. Tawk M, Goodrich S, Kinasewitz G, Orr W. The effect of 1 week of continuous positive airway pressure treatment in obstructive sleep apnea patients with concomitant gastroesophageal reflux. *Chest*. 2006;130(4):1003-1008. doi:10.1378/chest.130.4.1003

For peer review only

13. Shepherd K, Ockelford J, Ganasan V, Holloway R, Hillman D, Eastwood P. Temporal Relationship Between Night-Time Gastroesophageal Reflux Events and Arousals From Sleep. *Am J Gastroenterol*. 2020;115(5):697-705. doi:10.14309/ajg.0000000000000627

14. Green BT, Broughton WA, O'Connor JB. Marked improvement in nocturnal gastroesophageal reflux in a large cohort of patients with obstructive sleep apnea treated with continuous positive airway pressure. *Arch Intern Med*. 2003;163(1):41-45. doi:10.1001/archinte.163.1.41

15. Tamanna S, Campbell D, Warren R, Ullah MI. Effect of CPAP therapy on symptoms of nocturnal gastroesophageal reflux among patients with obstructive sleep apnea. *Journal of Clinical Sleep Medicine*. 2016;12(9):1257-1261. doi:10.5664/jcsm.6126

16. Shepherd K, Hillman D, Holloway R, Eastwood P. Mechanisms of nocturnal gastroesophageal reflux events in obstructive sleep apnea. *Sleep and Breathing*. 2011;15(3):561-570. doi:10.1007/s11325-010-0404-x

17. Sharples L, Glover M, Clutterbuck-James A, et al. Clinical effectiveness and cost-effectiveness results from the randomised controlled Trial of Oral Mandibular Advancement Devices for Obstructive sleep apnoea–hypopnoea (TOMADO) and long-term economic analysis of oral devices and continuous positive airwa. *Health Technol Assess (Rockv)*. 2014;18(67). doi:10.3310/hta18670

18. Carlos Rejón-Parrilla J, Garau M, Sussex J. *Obstructive Sleep Apnoea Health Economics Report Consulting Report for the British Lung Foundation*.; 2014.

19. Guest JF, Helter MT, Morga A, Stradling JR. Cost-effectiveness of using continuous positive airway pressure in the treatment of severe obstructive sleep apnoea/hypopnoea syndrome in the UK. doi:10.1136/thx.2007.086454

20. Grant A, Wileman S, Ramsay C, et al. *Health Technology Assessment NIHR HTA Programme Www.Hta.Ac.Uk The Effectiveness and Cost-Effectiveness of Minimal Access Surgery amongst People with Gastro-Oesophageal Reflux Disease-a UK Collaborative Study. The Reflux Trial*. Vol 12.; 2008. www.hta.ac.uk

21. Sekhon M, Cartwright M, Francis JJ. Acceptability of healthcare interventions: an overview of reviews and development of a theoretical framework. doi:10.1186/s12913-017-2031-8

22. Ritchie J, Spencer L. *Qualitative Data Analysis for Applied Policy Research*. Routledge; 1994.

23. Gannon B. The Feasibility Study: A Health Economics Perspective. *Global & Regional Health Technology Assessment: Italian; Northern Europe and Spanish*. 2017;4(1):grhta.5000254. doi:10.5301/grhta.5000254

24. Edwards RT, McIntosh E. *Applied Health Economics for Public Health Practice and Research*. (Edwards RT, McIntosh E, eds.). Oxford University Press; 2019.

25. Drummond MF, Sculpher MJ, Claxton K, Stoddart GL, Torrance GW. *Methods for the Economic Evaluation of Health Care Programmes*. 4th ed. Oxford University Press; 2015.

For peer review only

26. Skivington K, Matthews L, Simpson SA, et al. A new framework for developing and evaluating complex interventions: Update of Medical Research Council guidance. *The BMJ*. 2021;374. doi:10.1136/bmj.n2061

27. Herdman M, Gudex • C, Lloyd • A, et al. Development and preliminary testing of the new five-level version of EQ-5D (EQ-5D-5L). doi:10.1007/s11136-011-9903-x

28. Grewal I, Lewis J, Flynn T, Brown J, Bond J, Coast J. Developing attributes for a generic quality of life measure for older people: Preferences or capabilities? *Soc Sci Med*. 2006;62(8):1891-1901. doi:10.1016/J.SOCSCIMED.2005.08.023

29. Al-Janabi H, Flynn TN, Coast J. Development of a self-report measure of capability wellbeing for adults: the ICECAP-A. doi:10.1007/s11136-011-9927-2

30. Tennant R, Hiller L, Fishwick R, et al. The Warwick-Edinburgh Mental Well-being Scale (WEMWBS): development and UK validation. *Health Qual Life Outcomes*. 2007;5(1):63. doi:10.1186/1477-7525-5-63

31. Angus C, Cookson R, Griffin S, Norheim OF, Culyer AJ. *Distributional Cost-Effectiveness Analysis: Quantifying Health Equity Impacts and Trade-Offs Costs and Health Effects*. Oxford University Press; 2020. Accessed February 15, 2023. www.oxfordmedicine.com

32. Husereau D, Drummond M, Augustovski F, et al. Consolidated Health Economic Evaluation Reporting Standards 2022 (CHEERS 2022) Statement: Updated Reporting Guidance for Health Economic Evaluations. *BMJ*. 2022;376:067975.

33. Vemer P, Corro Ramos I, van Voorn GAK, Al MJ, Feenstra TL. AdViSHE: A Validation-Assessment Tool of Health-Economic Models for Decision Makers and Model Users. *Pharmacoeconomics*. 2016;34(4):349-361. doi:10.1007/s40273-015-0327-2

34. Eldridge S, Chan C, Campbell M, Bond C, Hopewell S, Thanane L. CONSORT 2010 statement: extension to randomised pilot and feasibility trials. *BMJ*. 2016;355.

35. Kerr P, Shoenut JP, Millar T, Buckle P, Kryger MH. Nasal CPAP reduces gastroesophageal reflux in obstructive sleep apnea syndrome. *Chest*. 1992;101(6):1539-1544. doi:10.1378/chest.101.6.1539

36. Cocks K, Torgerson DJ. Sample size calculations for pilot randomized trials: A confidence interval approach. *J Clin Epidemiol*. 2013;66(2):197-201. doi:10.1016/j.jclinepi.2012.09.002

37. Lancaster GA, Dodd S, Williamson PR. Design and analysis of pilot studies: Recommendations for good practice. *J Eval Clin Pract*. 2004;10(2):307-312. doi:10.1111/j..2002.384.doc.x

For peer review only

For peer review only

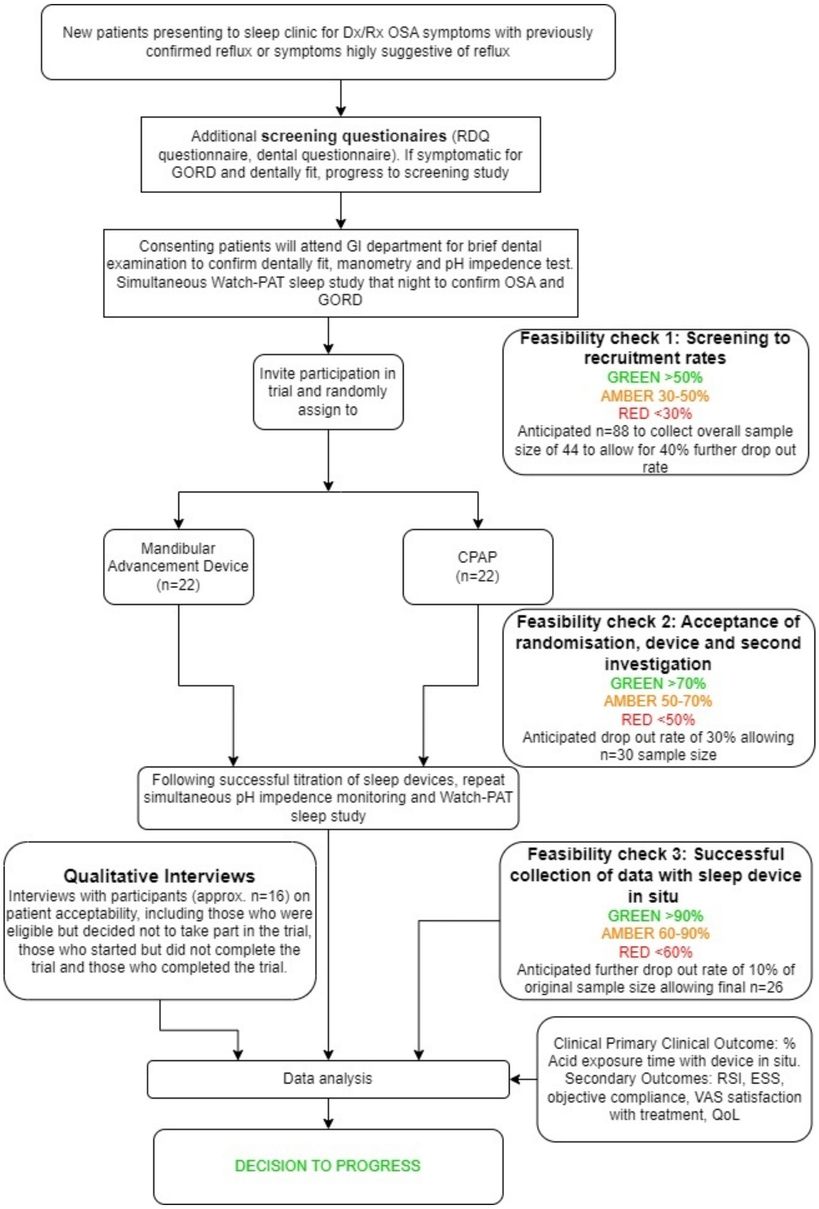

Study Flow Chart Demonstrating Feasibility Outcomes

229x339mm (72 x 72 DPI)

BMJ Open

A Single-Centre, Single-blinded, Randomised, Parallel group, Feasibility Study Protocol Investigating if Mandibular Advancement Device Treatment for Obstructive Sleep Apnoea can reduce Nocturnal Gastro-Oesophageal Reflux (MAD-Reflux trial).

Journal:	*BMJ Open*
Manuscript ID	bmjopen-2023-076661.R1
Article Type:	Protocol
Date Submitted by the Author:	10-Aug-2023
Complete List of Authors:	O'Toole, Saoirse; King's College London, ; University College Dublin,<br>Moazzez, Rebecca; University of the Pacific Arthur A Dugoni School of Dentistry<br>Wojewodka, Gabriella; King's College London, Oral and Clinical Research Unit<br>Zeki, Sebastian; Guy's and St Thomas' Hospitals NHS Trust<br>Jafari, Jafar; Guy's and St Thomas' Hospitals NHS Trust<br>Hope, Katherine; Hope2Sleep<br>Brand, Andrew; Bangor University, Statistics<br>Hoare, Zoe; Bangor University, North Wales Organisation for Randomised Trials in Health<br>Scott, Suzanne; Queen Mary University of London Wolfson Institute of Population Health<br>Doungsong, Kodchawan; Bangor University, Centre for Health Economics and Medicines Evaluation<br>Ezeofor, Victory; Bangor University,<br>Edwards, Rhiannon; Bangor University, Centre for Health Economics & Medicines Evaluation<br>Drakatos, Panagis; Guy's and St Thomas' Hospitals NHS Trust<br>Steier, Joerg; Guy`s & St Thomas` NHS Foundation Trust, Lane Fox Unit / Sleep Disorders Centre; King`s College London, School of Medicine
**Primary Subject Heading**:	Gastroenterology and hepatology
Secondary Subject Heading:	Dentistry and oral medicine, Respiratory medicine
Keywords:	Health economics < HEALTH SERVICES ADMINISTRATION & MANAGEMENT, SLEEP MEDICINE, GASTROENTEROLOGY, Feasibility Studies, ORAL MEDICINE

For peer review only - http://bmjopen.bmj.com/site/about/guidelines.xhtml

SCHOLARONE™
Manuscripts

A Single-Centre, Single-blinded, Randomised, Parallel group, Feasibility Study Protocol Investigating if Mandibular Advancement Device Treatment for Obstructive Sleep Apnoea can reduce Nocturnal Gastro-Oesophageal Reflux (MAD-Reflux trial).

Abstract

Introduction: Just under half of patients with obstructive sleep apnoea (OSA) also have gastro-oesophageal reflux disease (GORD). These conditions appear to be inter-related and Continual Positive Airway Pressure (CPAP) therapy, the gold standard treatment for OSA to prevent airway collapse, has been shown to reduce GORD. As the impact of Mandibular Advancement Devices (MADs), a second line therapy for OSA, on GORD has yet to be investigated,a feasibility study is needed prior to a definitive trial.

Methods: This will be a single-centre, single-blinded, tertiary-care based, interdisciplinary, parallel randomised controlled study. Potential OSA participants presenting to the sleep department will be pre-screened for GORD using validated questionnaires, consented and invited to receive simultaneous home sleep and oesophageal pH monitoring. Those with confirmed OSA and GORD (n=44) will be randomly allocated to receive either CPAP (n=22) or MAD therapy (n=22). Following successful titration and 3 weeks customisation period, participants will repeat the simultaneous sleep and oesophageal pH monitoring while wearing the device. The number of patients screened for recruitment, drop-out rates, patient feedback of the study protocol, costs of interventions and clinical information to inform a definitive study design, will be investigated.

Ethics and Dissemination: Health Research Authority approval has been obtained from the Nottingham 2 Research Ethics Committee, ref:22/EM/0157 and the trial has been registered on ISRCTN (https://doi.org/10.1186/ISRCTN16013232). Definitive findings about the feasibility of doing 24 hr pH oesophageal monitoring whilst doing a home sleep study will be disseminated via clinical and research networks facilitating valuable insights into the simultaneous management of both conditions.

Keywords: Obstructive sleep apnoea, gastro-oesophageal reflux, mandibular advancement devices, health economics, economic evaluation, process evaluation, feasibility study

Strengths and Limitations:

- This feasibility protocol is designed to gauge patient recruitment, acceptability and signal clinical efficacy for a definitive power calculation.

For peer review only - http://bmjopen.bmj.com/site/about/guidelines.xhtml

- The clinical trial is interdisciplinary involving dental, sleep and gastroenterology clinicians alongside patients, health psychologists, health economists and statisticians.
- Feasibility for health economic outcomes are also being assessed..
- Findings will inform a definitive trial rather than a clinical outcome.

Introduction

One billion people worldwide and 8 million people in the UK (24.5% of the population) are estimated to have Obstructive Sleep Apnoea (OSA)[1]. It is the most common sleep disorder, characterised by frequent pauses in breathing (apnoeas and hypopnoeas) during sleep due to airway blockage caused by intermittent relaxation of the throat muscles. Sleep becomes broken, so in the daytime patients complain of sleepiness, fatigue, and have other symptoms such as difficulty concentrating, memory impairment, feeling irritable and depressed [2]. Their own and their bed partner's quality of life is reduced [2]. Patients are at a higher risk of type 2 diabetes, high blood pressure, heart disease, strokes, and dying of all causes compared to people without OSA. This risk increases with OSA severity; with severe OSA, the risk of cardiac events and strokes is doubled and of cardiac death is tripled [3]. People with untreated OSA also put the public in danger as they have more road traffic accidents than people without OSA and compared to themselves once on treatment [4], [5]. The societal level-costs of undiagnosed OSA in the United States alone amounted to over $150 billion per year [6]. In Italy, OSA causes an economic burden ranging from €10.7 to €32 billion per year [7].

Gastro-Oesophageal Reflux Disease (GORD), defined by the Montreal consensus as "a condition that develops when the reflux of stomach contents causes troublesome symptoms and/or complications" with oesophageal/typical and extraoesophageal/ atypical symptoms [8] is also common in the UK. There is a strong association between obstructive sleep apnoea and GORD, partly due to common risk factors such as obesity, high alcohol consumption and diet but also due to the impact that apnoeic events have on the lower oesophageal sphincter [9]. A recent meta-analysis estimated that 45.2% of OSA patients had episodes of extra-oesophageal reflux independent of BMI [9]. Around 40-81% of patients suffering from GORD report that their symptoms occur during sleep [10]. There is a growing body of evidence that Continuous

For peer review only - http://bmjopen.bmj.com/site/about/guidelines.xhtml

Positive Airway Pressure (CPAP) therapy, the gold standard therapy for OSA [11], can reduce levels of gastro-oesophageal disease [12]–[15] by maintaining a patent airway, thus reducing intrathoracic pressure differentials. CPAP therapy increases the baseline lower oesophageal sphincter barrier pressure during sphincter relaxation and decreases the duration of sphincter relaxation [16]. Mandibular advancement devices (MADs), a second line therapy, also maintain a patent airway which may also have a similar impact on intrathoracic pressure differentials and duration of lower oesophageal sphincter relaxation. In addition, the greater compliance observed with MADs may mean that reflux is suppressed for a greater proportion of the night [17]. However, both theories remain untested to date. We do not have sufficient data to gauge an effect size for a definitive powered trial. We do not know if patients are willing to wear multiple devices and tests whilst trying to sleep and whether acceptability will influence the data. We are also unaware of the most appropriate quality of life measure to accurately assess patient impact and health economics. Therefore, a feasibility trial is needed prior to a definitive evaluation.

From a cost perspective, CPAP and MADs are both cost-effective treatments [18]. CPAP therapy has been shown to generate an incremental cost-effectiveness ratio (ICER) below £5,000 per quality-adjusted life-year (QALY) gained [19]. Using CPAP over a period of 14 years resulted in saving National Health Service (NHS) costs (nearly £1,000 per patient) and generated health benefits for people with OSA e.g. reduction in stroke risk and road traffic accidents [20].

In the UK, the yearly medication costs of GORD treatment are around £200 million for histamine receptor antagonists (H_2RAs) and £300 million for proton pump inhibitors (PPIs) [21]. The cost implication of the impact of CPAP therapy or MAD therapy on GORD has yet to be evaluated.

The overall aim of this project is to conduct a feasibility study to assess patient recruitment and tolerance of the trial. The secondary aims are to calculate an estimate of the primary outcome effect to determine the sample size needed for the definitive trial if the progression criteria are met, to determine the optimum measure of quality-of-life impact for this patient cohort.

Methods and Analysis

Study Design

For peer review only - http://bmjopen.bmj.com/site/about/guidelines.xhtml

This will be a single-centre, single-blinded, tertiary care based, interdisciplinary parallel 1:1 randomised controlled study. The trial has been registered on ISRCTN (reference 16013232, https://doi.org/10.1186/ISRCTN16013232).

Setting

Participants will be recruited from King's Health Partners sleep services at Guy's and St Thomas's Foundation Trust (GSTT, London, UK).

Outcomes:

Primary outcome measures

1. Percentage of approached patients who were screened for the trial measured using the percentage of patients eligible of those screened at Day 2 of the screening visit.

2. Percentage of eligible patients who were randomised measured using the percentage of screened patients who met the eligibility criteria versus those who were randomised at the randomisation timepoint.

3. Percentage of patients who completed the trial measured using number of patients who were randomised versus those who completed the trial at Day 2 of the final visit.

Primary clinical outcome to signal efficacy and inform a definitive power calculation.

1. Change in percentage acid contact time pH < 4 with the device in situ measured using nocturnal data from 24-hour pH monitoring at initial gastroenterology screening visit and follow-up gastroenterology assessment post therapy.

Secondary outcome measures

1. Patient acceptability of the trial measured using qualitative interviews throughout the trial.

2. Hours that device is worn during sleep measured using the average number of hours worn per night as detected from the output from the therapeutic device throughout the trial period.

3. To assess the most sensitive quality of life questionnaire prior to the full trial by recording EuroQol 5-dimension 5-level (EQ-5D-5L) [22], ICEpop CAPability measure for the adult population (ICECAP-A) [23], [24] and the Short Warwick–Edinburgh Mental Wellbeing Scale (SWEMWBS [25]) at baseline and post intervention.

4. Rating of both therapies measured using a Visual Analog Scale (VAS) at Day 2 of final assessment visit (trial completion).

5. Number of potential participants identified by the care team with and without screening of referral letters measured using number of participants identified on clinics before the pre-screening clinic and after the pre-screening.

For peer review only - http://bmjopen.bmj.com/site/about/guidelines.xhtml

6. Change in Reflux Symptom Index (RSI) [26] using changes detected in the RSI validated questionnaire at baseline and post intervention.

7. Change in Epworth Sleepiness Scale (ESS) [27] using changes detected in the ESS validated questionnaire at baseline and post intervention.

8. Change in cough using changes detected in the validated Leicester Cough Questionnaire [28] at baseline and post intervention.

9. To identify and measure indicative costs and outcomes and select suitable economic outcomes measured by documenting health resource use including intervention costs, appointment times and attending health care practitioners throughout the trial.

10. To determine the acceptability of the intervention and economic data collection methods measured using qualitative questions at the qualitative interview (various timepoints).

Participants and recruitment

Potential participants will be identified by the direct care team either by running a search text function to screen their referral letters or by their consultation in clinic. Patients referred for investigation of OSA, who also have a previously confirmed diagnosis of GORD with a 24 hour pH study or typical heartburn and/ or regurgitation >3 times per week and Reflux Disease Questionnaire (RDQ) questionnaire score of >50%, will be informed by their direct care team that they may be eligible to be included in the study and asked if they would like to speak to a research dentist or nurse about participation. The pre-screening questions for dental examination will ask patients if they have at least 10 teeth in each jaw, no loose teeth, fillings or caps or obvious holes in your teeth. Interested participants who meet the pre-screening criteria, will be provided with a patient information sheet of the entire trial and invited to have a dental screening during their gastroenterology appointment, followed by a combined home sleep study and 24-hour pH impedance oesophageal monitoring. It will be explained that participation in the trial will be dependent upon meeting the strict inclusion criteria but they will be reimbursed for the screening appointment.

Inclusion/Exclusion Criteria

Patients will be included in the study if they are:

1. Aged 18 or over,
2. Confirmed OSA with Apnoea-Hypopnoea Index (AHI) score between 10 and 50
3. Confirmed gastro-oesophageal reflux disease with greater than 6 percent of acid exposure time <pH 4 over 24 hours,

For peer review only - http://bmjopen.bmj.com/site/about/guidelines.xhtml

4. No previous CPAP or MAD therapy,
5. Sufficient healthy teeth to support a mandibular advancement device i.e. 10 teeth in each jaw, no periodontal pockets >5, no frank cavitation or loose crowns/bridges,
6. Willing and able to provide informed consent to the study.

Patients will be ineligible if:

1. Pregnant or breast feeding,
2. Unable or unwilling to stop GORD medication 2 days prior to assessment or unable to undergo manometry and pH impedance testing,
3. Known liver disease or oesophageal/gastric varices,
4. Previous surgery or intervention for reflux such as fundoplication which may preclude pH impedance testing,
5. Any previous treatment for oesophageal neoplasia,
6. Unable/unwilling to tolerate either a CPAP mask or a mandibular advancement device,
7. Medical history likely to impact on 24-hour impedance testing e.g bulimia nervosa,
8. Participation in other research within previous 30 days.

Study screening procedures

Following written informed consent by a trained member of the research team, participants will undergo assessments at a screening visit to ensure eligibility is met. Participants will be informed that proton pump inhibitors will need to be stopped 7 days prior and H2-receptor antagonists or antacids 48 hours prior to this visit. Participants will complete a reflux symptom index questionnaire, Epworth Sleepiness Scale questionnaire, Leicester Cough Questionnaire and several QoL questionnaires. A full dental exam will be conducted to ensure that participants are dentally fit to wear a MAD if allocated to this intervention. For the gastroenterology assessment, the patient will initially need to undergo high resolution manometry (HRM). Following local analgesia of the nares the catheter will be introduced trans-nasally and the patient instructed to drink water through a straw whilst the HRM catheter is advanced to the stomach. The HRM catheter depth will be adjusted to ensure manometric visual of the upper oesophageal sphincter (UOS), the gastro-oesophageal junction (GOJ) and gastric pressures. 10 single swallows of 5ml will be performed with each being 20 seconds apart. Each 5ml water swallow will be assessed in accordance with Chicago classification (version 3) using Manoview software (version 3) (Sierra Scientific Instruments). The HRM catheter will then be removed.

For peer review only - http://bmjopen.bmj.com/site/about/guidelines.xhtml

Patients will then undergo reflux monitoring using Sandhill Scientific multichannel impedance pH catheters (ZANBG-44) which are inserted trans-nasally after applying local anaesthesia (xylocaine). The dual pH sensors of the catheter will be positioned 5cm below and above the manometric lower oesophageal sphincter (LOS). The impedance sensors will be positioned above the LOS by 3cm, 5cm, 9cm, 15cm and 19cm. The data will be captured by ZepHrTM recording device.

For the 24 hours while the probe is inserted, the participant will be asked to complete a food diary as food and drink will impact on their reflux. Stopping reflux medication and the food diary are part of standard care for this procedure.

Following placement of the catheter, participants will be invited for their home sleep study. A type 3 sleep study device, the WatchPAT 200 (WP200; Itamar Medical Ltd., Caesarea, Israel) will be given in addition to comprehensive instructions on how to perform a home sleep study. A 24h number for technical support will be provided to the patient. After their overnight sleep study, they will return the following day to the gastroenterology department for removal of the probe and return of the WatchPAT.

The gastroenterology data will be captured by ZepHrTM recording device and data will be analysed using the BioVIEW Analysis software (5.7.1.0). The sleep study data will be analysed by a qualified sleep technician.

Randomisation Procedures

Participants who meet the inclusion/exclusion criteria will be allocated 1:1 using fixed-block randomisation by the purpose-built computer-generated randomisation service provided by King's Clinical Trials Unit (KCTU) to either the CPAP (n = 22) or the MAD (n = 22) arm. Once the participant identifier is entered into the system, an e-mail will be sent to the principal investigators and trial manager informing them of the allocation.

Interventions

The intervention will consist of either a MAD (n=22, SomnoMed Avant, Somnomed UK) with the dental sleep medicine department or CPAP (n=22, S8/S9, ResMed Ltd, Sydney, Australia) with the sleep medicine department.

Titration of Devices and Repeat Assessment

For peer review only - http://bmjopen.bmj.com/site/about/guidelines.xhtml

The CPAP mask will be delivered as standard of care and therefore may be different for each patient. The same CPAP therapy allocated to the participant will be used in the follow up investigation. Some adjustment of the devices may be needed to ensure comfort and efficacy of the device. This will be done as routine care and scheduled as needed.

Following successful titration for each device and a three-week customisation period, the 24-hour impedance monitoring and home sleep study will be repeated. As at visit 1, participants will be asked to cease their proton pump inhibitors, H2-receptor antagonists, or antacids. A dental examination will be carried out by the research dentist to ensure there have been no changes in the oral cavity. Participants will be asked to review their diet diary from the day they first did the test and repeat what they ate or drank as closely as possible for the day. That afternoon the participant will attend the oesophageal physiology laboratory. Participants will repeat the reflux symptom index questionnaire, Epworth Sleepiness Scale questionnaire, Leicester Cough Questionnaire and a QoL questionnaire. The pH impedance testing and Watch-PAT testing will be repeated as described above. Compliance levels for the MAD and CPAP therapy on the same night will be obtained.

Upon returning the next day to have their pH probe removed and to return the Watch-PAT, participants will be asked to complete a questionnaire about their views in taking part in the study.

Blinding

This is a single-blinded study. The statistician is the only member of the team who can be blinded to the allocation. The database has been created such that the intervention is not known to the statistician when performing the analysis. The patient and clinician cannot be blinded to the group allocation due to the nature of the therapeutic devices used. However, the objective, computer-generated clinical measurement outcome in for the definitive trial is less likely to be subject to bias.

Data collection and management

Clinical, demographic and questionnaire data will be collected on case report forms. Clinical data from manometry, impedance testing and sleep study will be obtained through patient electronic records and recorded on case report forms. Paper forms will be used, and data will be recorded on electronic case report forms held in a database using MACRO 4.0 (Infermed) and maintained

For peer review only - http://bmjopen.bmj.com/site/about/guidelines.xhtml

by the KCTU. Data checks will be performed by the trial manager and the trial statistician. The statistician and principal investigator will have access to the full trial dataset at the end of the trial.

Qualitative Interviews

To enhance our understanding of acceptability of recruitment processes, screening procedures, and of taking part in the trial we will conduct telephone interviews with participants (approx. n=16) over the course of the trial. We will use a sampling matrix to include patients who elected not to take part, eligible patients who were screened but did not take part in the trial, patients who could not tolerate the device or discontinued the trial for other reasons, and those who completed the trial.

Using the Theoretical Framework of Acceptability [29] to underpin the interview guide, participants will be asked about different domains of acceptability (e.g. affective attitude, burden, coherence, perceived effectiveness) for each element of the trial, including recruitment, randomisation, appointments and procedures, use of devices. Participants will also be asked about barriers to trial participation, potential improvements, and how to create advocacy for the trial amongst stakeholders. Participants will receive an additional £25 if they take part in this 25-minute interview. The interviews will be audio-recorded, anonymised and professionally transcribed verbatim, in preparation for Framework Analysis [30] whereby a thematic framework is developed and applied to the transcripts, allowing systematic analysis and interpretation of the qualitative data. Data will be managed in NVivo.

Health Economic Measures

During the feasibility phase, the best way of collecting relevant service costs, and patient borne costs, from both an NHS and a wider societal perspective will be investigated to inform a future full trial context [31]–[33]. We will consider to what extent this intervention constitutes a complex intervention, drawing on guidance from the UK Medical Research Council (MRC) for the guidance for complex interventions [34]. In the context of the patient, we will pilot the (EQ-5D-5L) [22], ICECAP-A [23], [24] and the Short Warwick–Edinburgh Mental Wellbeing Scale (SWEMWBS [25]) which could be used in a full RCT to calculate cost per Quality Adjusted Life Years (QALYs) and wider outcomes. We will determine if these are sufficiently sensitive to measure change in the patient group. We will also determine the appropriate deterministic sensitivity analysis in a full economic analysis in this patient group. We will pay attention to distributive cost-effectiveness analysis opportunities in a full trial [35].

For peer review only - http://bmjopen.bmj.com/site/about/guidelines.xhtml

Our base-case analysis will explore the likely total costs (intervention costs and medical costs) and outcomes (health resource service use and health-related quality of life) of using MADs compared to CPAP therapy in people with OSA who have nocturnal GORD in a future full trial. We will also explore if these outcome measures accurately reflect the conditions of the patients.

We will write a report to the funder and papers for peer-review publication following the Consolidated Health Economic Evaluation Reporting Standards (CHEERS) statement [36] and the Assessment of the Validation Status of Health-Economic Decision Model (AdVISHE) [37]. The health economic feasibility study will be reported in accordance with the Consolidated Standards of Reporting Trials (CONSORT) extension [38].

A full schedule of events is given in Supplementary Table 1 and the patient flow diagram with feasibility outcomes is illustrated in Figure 1.

Adverse events

All adverse events (AEs) will be recorded from the time of randomisation. AEs will be classified according to severity and whether related to the study intervention. All Serious Adverse Events SAEs will be reported immediately by the Chief Investigator and, no later than 24hrs, to the Research & Development office (Sponsor) and main Research Ethics Committee.

Sample Size

There are several studies investigating change in % acid exposure from baseline whilst wearing the CPAP device. Samples sizes suggested from current data based on change in total acid were between 86 to 168 per group (excluding any attrition)[12], [39]. Using the smallest effect size published (Tawk et al.[12]), assuming a one-sided non-inferiority design with a margin of 4.15 (% total acid) together with a SD of 10.8 with a power of 90% and alpha of 2.5% for a definitive study, this would require a sample of 288 (without attrition). Considering the likelihood of the main study finding this an effect of this size within an 80% confidence interval, then an approach taken by Cocks and Torgerson [40] requires a feasibility sample equivalent to approximately 9% of the proposed definitive sample requiring 26 participants. Accommodating an overall attrition rate, including tolerance to the devices, of 40% requires a sample of 44 to be randomised. A sample of 44 will also give us a 95% confidence interval of +/- 14% around the attrition rate.

Statistical Analysis

Baseline characteristics will be summarized for all participants within the trial arms.

For peer review only - http://bmjopen.bmj.com/site/about/guidelines.xhtml

The feasibility outcomes include:

1. Percentage of screened patients who were eligible for the trial.
2. Percentage of eligible patients who agreed to participate.
3. Percentage of patients who completed the trial.

We will then assess one clinical outcome to assess signal of efficacy which is the change in percentage acid contact time pH < 4 with the device in situ. Participants' uptake of and adherence to both CPAP and MAD, as well as follow-up rates, will be summarized and presented as percentages. Although determining differences in clinical outcomes between the arms is not the primary purpose of this feasibility study, comparisons will be undertaken to investigate the feasibility of studying these outcomes and to calculate potential estimates and 95% confidence intervals. As recommended in guidelines for good practice for the analysis of pilot studies [41], the focus of the results will be on the estimates of the treatments rather than statistical significance and as such no hypothesis testing will be undertaken. Differences between the two comparison groups will be presented in the form of an unadjusted mean difference for continuous outcomes, and an odds ratio for binary outcomes, with their associated 95% confidence intervals. These comparisons will be made on an intention to treat basis with consideration given to per protocol analysis as sensitivity. While every effort will be made to minimise missing data, assessment of the levels of missing data will indicate suitability of measures to be continued into the definitive trial.

Discussion

Given the increasing prevalence of both OSA and GORD, it is important to further investigate the inter-relationship between the conditions and the impact of OSA treatment on GORD. While there is evidence that CPAP reduces GORD, the effect size is unclear, and the impact of MADs on GORD is entirely un-investigated.

This feasibility trial is designed as a first step to provide this important information for patients deciding on treatment choices and the prescribing sleep clinician. This trial will give us insights into the feasibility for testing patients, patient experience, the mechanism of action and the overall effect. There were several items for consideration when developing the study design. Firstly, we chose to use a level 2 sleep study rather than polysomnography. This was because a level 2 device is sufficient for diagnosis of OSAS provided no other respiratory/sleep pathologies are suspected. At the time of recruitment, all participants had been assessed by their managing

For peer review only - http://bmjopen.bmj.com/site/about/guidelines.xhtml

consultant and diagnosed as having suspect OSAS. The primary outcome of this study is a gastroenterology outcome and AHI is used solely as the inclusion criteria. Furthermore, the final assessment night for the patient will necessitate them to wear a sleep study device, a nasal pH monitor and their MAD or CPAP therapeutic intervention. We thought this would be easier for the patient if done at home. Lastly, we would like the definitive trial to be a multi-centre trial. Not all centres will have capacity for polysomnography. For these reasons we deemed that a Watch-PAT was appropriate. We have also considered items such as the interference of the nasal catheter with the CPAP mask. This has been extensively discussed with our PPI group some of whom have already undergone 24hour pH monitoring while wearing their CPAP. The mask seal can be maintained with strategic placement of the nasal catheter and tape. There is also precedent for this in previous studies [12], [16]. However, the feasibility of this will be explored.

We will also explore health economic measures to best capture the cost of services versus the value obtained for patients and health care providers. From a UK National Health Service perspective, both CPAP and MADs are reported to be similarly cost-effective treatments [18]. This may change once their efficacy on the management of nocturnal gastro-oesophageal reflux is assessed. Investigation into the health economic feasibility for other funding systems is beyond the scope of this initial feasibility paper. However, we will be informing on the best measures to capture health economic outcomes which can then be used in different healthcare funding environments.

Patient and public involvement statement

The trial was designed to generate evidence for patients with both OSA and GORD who are deciding on their treatment options. Our patient co-applicant is highly active within the OSA community with both feedback and dissemination means at a national level (Hope2Sleep Charity). We have an active PPI group who were also involved in the review and development of study protocol and patient information sheets. Patient advocacy is an important part of our oversight committee and our patient co-applicant is in charge of ensuring the patient perspective is presented throughout the duration of the trial lifecycle. All findings from the study will be made available for dissemination to the public.

Ethics and Dissemination: Health Research Authority approval has been obtained from the Nottingham 2 Research Ethics Committee, ref:22/EM/0157 and the trial has been registered on ISRCTN (https://doi.org/10.1186/ISRCTN16013232) with access to the full protocol. Definitive findings about the feasibility of doing 24 hr pH oesophageal monitoring whilst doing a home sleep

For peer review only - http://bmjopen.bmj.com/site/about/guidelines.xhtml

study will be disseminated via clinical and research networks facilitating valuable insights into the simultaneous management of both conditions.

Authors' Contributions

SOT, RM, ZS, JJ, GW, KH, AB, ZH, SS, RTE, VE, KD, PD and JS contributed to the conception and design of the study. SOT drafted the manuscript. All authors critically revised the manuscript. All authors gave their final approval and agree to be accountable for all aspects of the work.

Conflicts of Interest

The authors have no conflicts of interest to declare

Funding Statement

This project is funded by the National Institute for Health and Care Research (NIHR) under its Research for Patient Benefit (RfPB) Programme (Grant Reference Number NIHR202744). The views expressed are those of the authors and not necessarily those of the NIHR or the Department of Health and Social Care. This study is sponsored by Guy's and St Thomas's trust and King's College London. The funder and sponsor did not have any authority over the intellectual development and decision to submit this paper for publication.

Acknowledgement

Thanks to Dr Catherine Lawrence for reading support for RTE.

Figure Legend

Figure 1. Study flow chart showing feasibility outcomes

Supplementary Table 1. Schedule of events, interventions and assessments for the MAD Reflux Trial according to Spirit Guidelines

References

[1] A. J. Wimms *et al.*, "Continuous positive airway pressure versus standard care for the treatment of people with mild obstructive sleep apnoea (MERGE): a multicentre, randomised controlled trial," *Lancet Respir Med*, vol. 8, no. 4, pp. 349–358, Apr. 2020, doi: 10.1016/S2213-2600(19)30402-3.

[2] C. A. Moyer, S. S. Sonnad, S. L. Garetz, J. I. Helman, and R. D. Chervin, "Quality of life in obstructive sleep apnea: A systematic review of the literature," *Sleep Medicine*, vol. 2, no. 6. Sleep Med, pp. 477–491, 2001. doi: 10.1016/S1389-9457(01)00072-7.

For peer review only - http://bmjopen.bmj.com/site/about/guidelines.xhtml

[3] C. Xie, R. Zhu, Y. Tian, and K. Wang, “Association of obstructive sleep apnoea with the risk of vascular outcomes and all-cause mortality: A meta-analysis,” *BMJ Open*, vol. 7, no. 12, Dec. 2017, doi: 10.1136/bmjopen-2016-013983.

[4] M. Karimi, J. Hedner, H. Häbel, O. Nerman, and L. Grote, “Sleep apnea related risk of motor vehicle accidents is reduced by continuous positive airway pressure: Swedish traffic accident registry data,” *Sleep*, vol. 38, no. 3, pp. 341–349, Mar. 2015, doi: 10.5665/sleep.4486.

[5] S. Garbarino *et al.*, “Sleep apnea, sleep debt and daytime sleepiness are independently associated with road accidents. A cross-sectional study on truck drivers,” *PLoS One*, vol. 11, no. 11, Nov. 2016, doi: 10.1371/journal.pone.0166262.

[6] A. N. Natsky, A. Vakulin, C. L. C. Coetzer, R. D. McEvoy, R. J. Adams, and B. Kaambwa, “Economic evaluation of diagnostic sleep studies for obstructive sleep apnoea: a systematic review protocol,” *Syst Rev*, vol. 10, no. 1, Dec. 2021, doi: 10.1186/s13643-021-01651-3.

[7] L. Borsoi, P. Armeni, G. Donin, F. Costa, and L. Ferini-Strambi, “The invisible costs of obstructive sleep apnea (OSA): Systematic review and cost-of-illness analysis,” *PLoS ONE*, vol. 17, no. 5 May. Public Library of Science, May 01, 2022. doi: 10.1371/journal.pone.0268677.

[8] N. Vakil *et al.*, “The Montreal definition and classification of gastroesophageal reflux disease: A global evidence-based consensus,” *American Journal of Gastroenterology*, vol. 101, no. 8. pp. 1900–1920, 2006. doi: 10.1111/j.1572-0241.2006.00630.x.

[9] G. Magliulo *et al.*, “Laryngopharyngeal reflux in obstructive sleep apnoea patients: Literature review and meta-analysis,” *American Journal of Otolaryngology - Head and Neck Medicine and Surgery*, vol. 39, no. 6. W.B. Saunders, pp. 776–780, Nov. 01, 2018. doi: 10.1016/j.amjoto.2018.09.006.

[10] W. C. Orr, “Review article: Sleep-related gastro-oesophageal reflux as a distinct clinical entity,” *Alimentary Pharmacology and Therapeutics*, vol. 31, no. 1. John Wiley & Sons, Ltd, pp. 47–56, Jan. 01, 2010. doi: 10.1111/j.1365-2036.2009.04124.x.

[11] NICE guideline [NG202], “Obstructive sleep apnoea/ hypopnoea syndrome and obesity hypoventilation syndrome in over 16s NICE guideline,” Aug. 2021. [Online]. Available: www.nice.org.uk/guidance/ng202

[12] M. Tawk, S. Goodrich, G. Kinasewitz, and W. Orr, “The effect of 1 week of continuous positive airway pressure treatment in obstructive sleep apnea patients with concomitant gastroesophageal reflux,” *Chest*, vol. 130, no. 4, pp. 1003–1008, 2006, doi: 10.1378/chest.130.4.1003.

[13] K. Shepherd, J. Ockelford, V. Ganasan, R. Holloway, D. Hillman, and P. Eastwood, “Temporal Relationship Between Night-Time Gastroesophageal Reflux Events and Arousals From Sleep,” *Am J Gastroenterol*, vol. 115, no. 5, pp. 697–705, 2020, doi: 10.14309/ajg.0000000000000627.

[14] B. T. Green, W. A. Broughton, and J. B. O’Connor, “Marked improvement in nocturnal gastroesophageal reflux in a large cohort of patients with obstructive sleep apnea treated with

For peer review only - http://bmjopen.bmj.com/site/about/guidelines.xhtml

continuous positive airway pressure," *Arch Intern Med*, vol. 163, no. 1, pp. 41–45, 2003, doi: 10.1001/archinte.163.1.41.

[15] S. Tamanna, D. Campbell, R. Warren, and M. I. Ullah, "Effect of CPAP therapy on symptoms of nocturnal gastroesophageal reflux among patients with obstructive sleep apnea," *Journal of Clinical Sleep Medicine*, vol. 12, no. 9, pp. 1257–1261, 2016, doi: 10.5664/jcsm.6126.

[16] K. Shepherd, D. Hillman, R. Holloway, and P. Eastwood, "Mechanisms of nocturnal gastroesophageal reflux events in obstructive sleep apnea," *Sleep and Breathing*, vol. 15, no. 3, pp. 561–570, Sep. 2011, doi: 10.1007/s11325-010-0404-x.

[17] M. Schwartz, L. Acosta, Y. L. Hung, M. Padilla, and R. Enciso, "Effects of CPAP and mandibular advancement device treatment in obstructive sleep apnea patients: a systematic review and meta-analysis," *Sleep and Breathing*, vol. 22, no. 3. Springer Verlag, pp. 555–568, Sep. 01, 2018. doi: 10.1007/s11325-017-1590-6.

[18] L. Sharples *et al.*, "Clinical effectiveness and cost-effectiveness results from the randomised controlled Trial of Oral Mandibular Advancement Devices for Obstructive sleep apnoea–hypopnoea (TOMADO) and long-term economic analysis of oral devices and continuous positive airwa," *Health Technol Assess (Rockv)*, vol. 18, no. 67, 2014, doi: 10.3310/hta18670.

[19] J. Carlos Rejón-Parrilla, M. Garau, and J. Sussex, "Obstructive Sleep Apnoea Health Economics Report Consulting Report for the British Lung Foundation," 2014.

[20] J. F. Guest, M. T. Helter, A. Morga, and J. R. Stradling, "Cost-effectiveness of using continuous positive airway pressure in the treatment of severe obstructive sleep apnoea/hypopnoea syndrome in the UK", doi: 10.1136/thx.2007.086454.

[21] A. Grant *et al.*, "Health Technology Assessment NIHR HTA Programme www.hta.ac.uk The effectiveness and cost-effectiveness of minimal access surgery amongst people with gastro-oesophageal reflux disease-a UK collaborative study. The reflux trial," 2008. [Online]. Available: www.hta.ac.uk

[22] M. Herdman *et al.*, "Development and preliminary testing of the new five-level version of EQ-5D (EQ-5D-5L)", doi: 10.1007/s11136-011-9903-x.

[23] I. Grewal, J. Lewis, T. Flynn, J. Brown, J. Bond, and J. Coast, "Developing attributes for a generic quality of life measure for older people: Preferences or capabilities?," *Soc Sci Med*, vol. 62, no. 8, pp. 1891–1901, Apr. 2006, doi: 10.1016/J.SOCSCIMED.2005.08.023.

[24] H. Al-Janabi, T. N. Flynn, and J. Coast, "Development of a self-report measure of capability wellbeing for adults: the ICECAP-A", doi: 10.1007/s11136-011-9927-2.

[25] R. Tennant *et al.*, "The Warwick-Edinburgh Mental Well-being Scale (WEMWBS): development and UK validation," *Health Qual Life Outcomes*, vol. 5, no. 1, p. 63, 2007, doi: 10.1186/1477-7525-5-63.

[26] P. C. Belafsky, G. N. Postma, and J. A. Koufman, "Validity and reliability of the reflux symptom index (RSI)," *Journal of Voice*, vol. 16, no. 2, pp. 274–277, 2002, doi: 10.1016/S0892-1997(02)00097-8.

For peer review only - http://bmjopen.bmj.com/site/about/guidelines.xhtml

[27] M. W. Johns, “A new method for measuring daytime sleepiness: The Epworth sleepiness scale,” *Sleep*, vol. 14, no. 6, pp. 540–545, 1991, doi: 10.1093/sleep/14.6.540.

[28] S. S. Birring, B. Prudon, A. J. Carr, S. J. Singh, L. Morgan, and I. D. Pavord, “Development of a symptom specific health status measure for patients with chronic cough: Leicester Cough Questionnaire (LCQ),” *Thorax*, vol. 58, no. 4, pp. 339–343, 2003, doi: 10.1136/thorax.58.4.339.

[29] M. Sekhon, M. Cartwright, and J. J. Francis, “Acceptability of healthcare interventions: an overview of reviews and development of a theoretical framework”, doi: 10.1186/s12913-017-2031-8.

[30] J. Ritchie and L. Spencer, *Qualitative Data Analysis for Applied Policy Research*. London: Routledge, 1994.

[31] B. Gannon, “The Feasibility Study: A Health Economics Perspective,” *Global & Regional Health Technology Assessment: Italian; Northern Europe and Spanish*, vol. 4, no. 1, p. grhta.5000254, Jan. 2017, doi: 10.5301/grhta.5000254.

[32] R. T. Edwards and E. McIntosh, *Applied Health Economics for Public Health Practice and Research*. Oxford University Press, 2019.

[33] M. F. Drummond, M. J. Sculpher, K. Claxton, G. L. Stoddart, and G. W. Torrance, *Methods for the Economic Evaluation of Health Care Programmes*, 4th ed. Oxford University Press, 2015.

[34] K. Skivington *et al.*, “A new framework for developing and evaluating complex interventions: Update of Medical Research Council guidance,” *The BMJ*, vol. 374, Sep. 2021, doi: 10.1136/bmj.n2061.

[35] C. Angus, R. Cookson, S. Griffin, O. F. Norheim, and A. J. Culyer, *Distributional Cost-Effectiveness Analysis: Quantifying Health Equity Impacts and Trade-Offs Costs and health effects*. Oxford University Press, 2020. Accessed: Feb. 15, 2023. [Online]. Available: www.oxfordmedicine.com

[36] D. Husereau *et al.*, “Consolidated Health Economic Evaluation Reporting Standards 2022 (CHEERS 2022) Statement: Updated Reporting Guidance for Health Economic Evaluations,” *BMJ*, vol. 376. p. 067975, 2022.

[37] P. Vemer, I. Corro Ramos, G. A. K. van Voorn, M. J. Al, and T. L. Feenstra, “AdViSHE: A Validation-Assessment Tool of Health-Economic Models for Decision Makers and Model Users,” *Pharmacoeconomics*, vol. 34, no. 4, pp. 349–361, 2016, doi: 10.1007/s40273-015-0327-2.

[38] S. Eldridge, C. Chan, M. Campbell, C. Bond, S. Hopewell, and L. Thanane, “CONSORT 2010 statement: extension to randomised pilot and feasibility trials,” *BMJ*, vol. 355, 2016.

[39] P. Kerr, J. P. Shoenut, T. Millar, P. Buckle, and M. H. Kryger, “Nasal CPAP reduces gastroesophageal reflux in obstructive sleep apnea syndrome,” *Chest*, vol. 101, no. 6, pp. 1539–1544, Jun. 1992, doi: 10.1378/chest.101.6.1539.

[40] K. Cocks and D. J. Torgerson, “Sample size calculations for pilot randomized trials: A confidence interval approach,” *J Clin Epidemiol*, vol. 66, no. 2, pp. 197–201, Feb. 2013, doi: 10.1016/j.jclinepi.2012.09.002.

For peer review only - http://bmjopen.bmj.com/site/about/guidelines.xhtml

[41] G. A. Lancaster, S. Dodd, and P. R. Williamson, “Design and analysis of pilot studies: Recommendations for good practice,” *J Eval Clin Pract*, vol. 10, no. 2, pp. 307–312, 2004, doi: 10.1111/j..2002.384.doc.x.

For peer review only

For peer review only - http://bmjopen.bmj.com/site/about/guidelines.xhtml

For peer review only

For peer review only - http://bmjopen.bmj.com/site/about/guidelines.xhtml

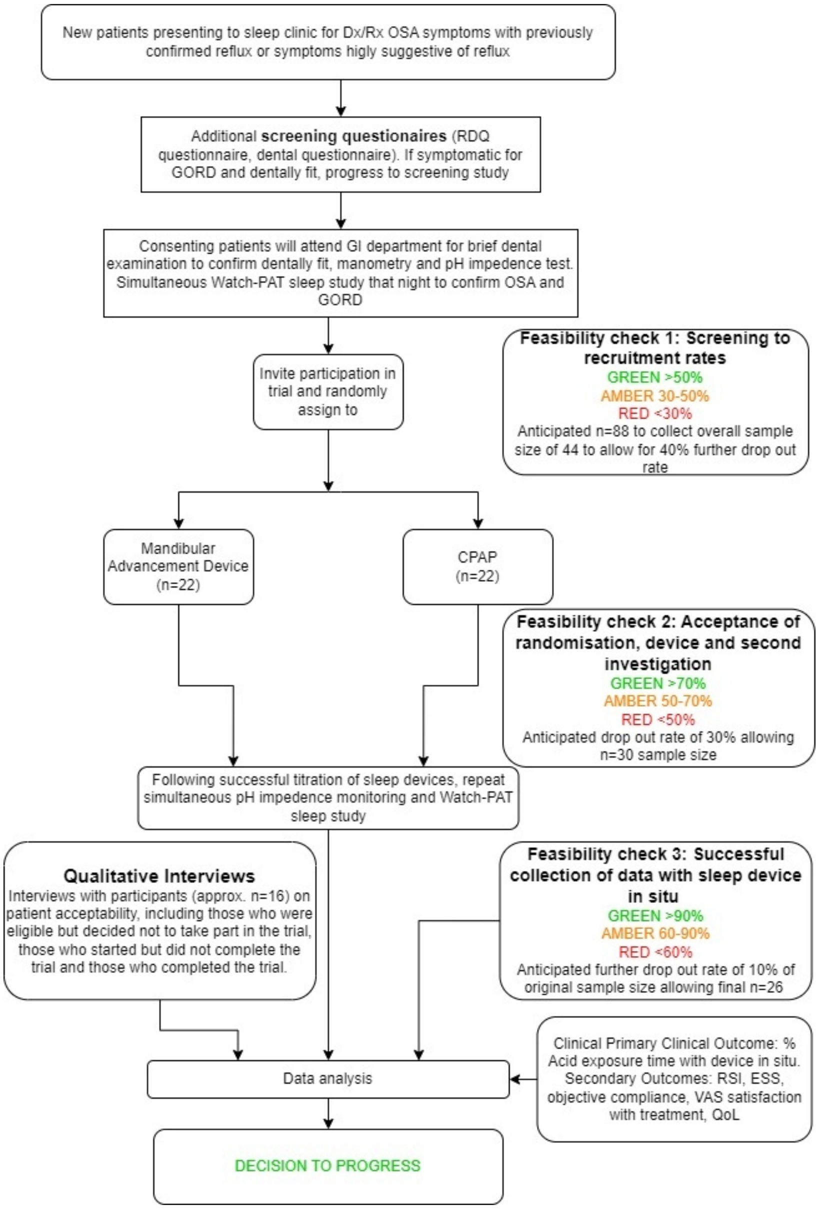

Study Flow Chart Demonstrating Feasibility Outcomes

229x339mm (72 x 72 DPI)

		Pre-Screening Visit	Visit 1: Screening visit		Visit 2 Intervention			Visit 3 or more	Final assessment visit	
					CPAP	MAD				
			Day 1	Day 2		App 1	App 2		Day 1	Day 2
**ENROLMENT**	Eligibility screen	x								
	Patient information sheet given and consent for additional screening procedures	x								
	RDQ and dental screen	x								
	Advice re reflux meds and recording diet prior to screening/final appointment	x						x		
	Full written informed consent		x							
	Dental examination, manometry and placement of pH catheter		x						x	
	Receive Watch-PAT home sleep monitoring		x						x	
	Return sleep monitoring device and remove pH catheter			x						x
	24h food intake recording while pH probe is placed		x	x					x	x
	Eligibility confirmed and randomisation			x						

For peer review only

**INTERVENTIONS**	CPAP									
	CPAP mask fitted and instructions given				x					
	MAD									
	Digital oral impressions taken for MAD and delivered 3 weeks later					x	x			
	Device titration appointments							x		
**ASSESSMENTS**	Documenting numbers for feasibility outcomes on those approached, screened and completed the trial	x		x						x
	Qualitative Interviews about participant views in taking part in the study	x		x						x
	RSI, ESS, Leicester Cough Questionnaire, QoL questionnaires		x						x	
	Change in percentage acid contact time pH < 4 with the device in situ									x
	Hours that device is worn during sleep									x

Table 1. Schedule of events, interventions and assessments for the MAD Reflux Trial according to Spirit Guidelines

For peer review only

Reporting checklist for protocol of a clinical trial.

Based on the SPIRIT guidelines.

Instructions to authors

Complete this checklist by entering the page numbers from your manuscript where readers will find each of the items listed below.

Your article may not currently address all the items on the checklist. Please modify your text to include the missing information. If you are certain that an item does not apply, please write "n/a" and provide a short explanation.

Upload your completed checklist as an extra file when you submit to a journal.

In your methods section, say that you used the SPIRITreporting guidelines, and cite them as:

Chan A-W, Tetzlaff JM, Gøtzsche PC, Altman DG, Mann H, Berlin J, Dickersin K, Hróbjartsson A, Schulz KF, Parulekar WR, Krleža-Jerić K, Laupacis A, Moher D. SPIRIT 2013 Explanation and Elaboration: Guidance for protocols of clinical trials. BMJ. 2013;346:e7586

		Reporting Item	Page Number
**Administrative information**			
Title	#1	Descriptive title identifying the study design, population, interventions, and, if applicable, trial acronym	1

For peer review only

Trial registration	#2a	Trial identifier and registry name. If not yet registered, name of intended registry	1
Trial registration: data set	#2b	n/a	
Protocol version	#3	Date and version identifier	Supplementary file
Funding	#4	Sources and types of financial, material, and other support	13
Roles and responsibilities: contributorship	#5a	Names, affiliations, and roles of protocol contributors	1
Roles and responsibilities: sponsor contact information	#5b	Name and contact information for the trial sponsor	1
Roles and responsibilities: sponsor and funder	#5c	Role of study sponsor and funders, if any, in study design; collection, management, analysis, and interpretation of data; writing of the report; and the decision to submit the report for publication, including whether they will have ultimate authority over any of these activities	13

For peer review only

Roles and responsibilities: committees	#5d	Composition, roles, and responsibilities of the coordinating centre, steering committee, endpoint adjudication committee, data management team, and other individuals or groups overseeing the trial, if applicable (see Item 21a for data monitoring committee)	n/a
**Introduction**			
Background and rationale	#6a	Description of research question and justification for undertaking the trial, including summary of relevant studies (published and unpublished) examining benefits and harms for each intervention	2-3
Background and rationale: choice of comparators	#6b	Explanation for choice of comparators	2-3
Objectives	#7	Specific objectives or hypotheses	4
Trial design	#8	Description of trial design including type of trial (eg, parallel group, crossover, factorial, single group), allocation ratio, and framework (eg, superiority, equivalence, non-inferiority, exploratory)	4
**Methods: Participants, interventions, and outcomes**			

For peer review only

Study setting	#9	Description of study settings (eg, community clinic, academic hospital) and list of countries where data will be collected. Reference to where list of study sites can be obtained	4
Eligibility criteria	#10	Inclusion and exclusion criteria for participants. If applicable, eligibility criteria for study centres and individuals who will perform the interventions (eg, surgeons, psychotherapists)	6
Interventions: description	#11a	Interventions for each group with sufficient detail to allow replication, including how and when they will be administered	8
Interventions: modifications	#11b	Criteria for discontinuing or modifying allocated interventions for a given trial participant (eg, drug dose change in response to harms, participant request, or improving / worsening disease)	10
Interventions: adherance	#11c	Strategies to improve adherence to intervention protocols, and any procedures for monitoring adherence (eg, drug tablet return; laboratory tests)	n/a
Interventions: concomitant care	#11d	Relevant concomitant care and interventions that are permitted or prohibited during the trial	n/a
Outcomes	#12	Primary, secondary, and other outcomes, including the specific measurement variable (eg, systolic blood pressure), analysis metric (eg, change from	4-5

For peer review only

		baseline, final value, time to event), method of aggregation (eg, median, proportion), and time point for each outcome. Explanation of the clinical relevance of chosen efficacy and harm outcomes is strongly recommended	
Participant timeline	#13	Time schedule of enrolment, interventions (including any run-ins and washouts), assessments, and visits for participants. A schematic diagram is highly recommended (see Figure)	13-14
Sample size	#14	Estimated number of participants needed to achieve study objectives and how it was determined, including clinical and statistical assumptions supporting any sample size calculations	10
Recruitment	#15	Strategies for achieving adequate participant enrolment to reach target sample size	n/a
**Methods: Assignment of interventions (for controlled trials)**			
Allocation: sequence generation	#16a	Method of generating the allocation sequence (eg, computer-generated random numbers), and list of any factors for stratification. To reduce	7-8

For peer review only

		predictability of a random sequence, details of any planned restriction (eg, blocking) should be provided in a separate document that is unavailable to those who enrol participants or assign interventions	
Allocation concealment mechanism	#16b	Mechanism of implementing the allocation sequence (eg, central telephone; sequentially numbered, opaque, sealed envelopes), describing any steps to conceal the sequence until interventions are assigned	7-8
Allocation: implementation	#16c	Who will generate the allocation sequence, who will enrol participants, and who will assign participants to interventions	7-8
Blinding (masking)	#17a	Who will be blinded after assignment to interventions (eg, trial participants, care providers, outcome assessors, data analysts), and how	8
Blinding (masking): emergency unblinding	#17b	If blinded, circumstances under which unblinding is permissible, and procedure for revealing a participant's allocated intervention during the trial	8
**Methods: Data collection, management, and analysis**			

For peer review only

Data collection plan	#18a	Plans for assessment and collection of outcome, baseline, and other trial data, including any related processes to promote data quality (eg, duplicate measurements, training of assessors) and a description of study instruments (eg, questionnaires, laboratory tests) along with their reliability and validity, if known. Reference to where data collection forms can be found, if not in the protocol	9
Data collection plan: retention	#18b	Plans to promote participant retention and complete follow-up, including list of any outcome data to be collected for participants who discontinue or deviate from intervention protocols	9 and 17
Data management	#19	Plans for data entry, coding, security, and storage, including any related processes to promote data quality (eg, double data entry; range checks for data values). Reference to where details of data management procedures can be found, if not in the protocol	9
Statistics: outcomes	#20a	Statistical methods for analysing primary and secondary outcomes. Reference to where other details of the statistical analysis plan can be found, if not in the protocol	11

For peer review only

Statistics: additional analyses	#20b	Methods for any additional analyses (eg, subgroup and adjusted analyses)	11
Statistics: analysis population and missing data	#20c	Definition of analysis population relating to protocol non-adherence (eg, as randomised analysis), and any statistical methods to handle missing data (eg, multiple imputation)	11
**Methods: Monitoring**			
Data monitoring: formal committee	#21a	Composition of data monitoring committee (DMC); summary of its role and reporting structure; statement of whether it is independent from the sponsor and competing interests; and reference to where further details about its charter can be found, if not in the protocol. Alternatively, an explanation of why a DMC is not needed	n/a
Data monitoring: interim analysis	#21b	Description of any interim analyses and stopping guidelines, including who will have access to these interim results and make the final decision to terminate the trial	n/a
Harms	#22	Plans for collecting, assessing, reporting, and managing solicited and spontaneously reported adverse events and other unintended effects of trial interventions or trial conduct	10

For peer review only

Auditing	#23	Frequency and procedures for auditing trial conduct, if any, and whether the process will be independent from investigators and the sponsor	10
**Ethics and dissemination**			
Research ethics approval	#24	Plans for seeking research ethics committee / institutional review board (REC / IRB) approval	13
Protocol amendments	#25	Plans for communicating important protocol modifications (eg, changes to eligibility criteria, outcomes, analyses) to relevant parties (eg, investigators, REC / IRBs, trial participants, trial registries, journals, regulators)	13
Consent or assent	#26a	Who will obtain informed consent or assent from potential trial participants or authorised surrogates, and how (see Item 32)	6
Consent or assent: ancillary studies	#26b	Additional consent provisions for collection and use of participant data and biological specimens in ancillary studies, if applicable	n/a
Confidentiality	#27	How personal information about potential and enrolled participants will be collected, shared, and maintained in order to protect confidentiality before, during, and after the trial	9

For peer review only

Declaration of interests	#28	Financial and other competing interests for principal investigators for the overall trial and each study site	13
Data access	#29	Statement of who will have access to the final trial dataset, and disclosure of contractual agreements that limit such access for investigators	9
Ancillary and post trial care	#30	Provisions, if any, for ancillary and post-trial care, and for compensation to those who suffer harm from trial participation	n/a
Dissemination policy: trial results	#31a	Plans for investigators and sponsor to communicate trial results to participants, healthcare professionals, the public, and other relevant groups (eg, via publication, reporting in results databases, or other data sharing arrangements), including any publication restrictions	13
Dissemination policy: authorship	#31b	Authorship eligibility guidelines and any intended use of professional writers	13
Dissemination policy: reproducible research	#31c	Plans, if any, for granting public access to the full protocol, participant-level dataset, and statistical code	13

Appendices

For peer review only

Informed consent materials	#32	Model consent form and other related documentation given to participants and authorised surrogates	Supplementary material
Biological specimens	#33	Plans for collection, laboratory evaluation, and storage of biological specimens for genetic or molecular analysis in the current trial and for future use in ancillary studies, if applicable	n/a

None The SPIRIT Explanation and Elaboration paper is distributed under the terms of the Creative Commons Attribution License CC-BY-NC. This checklist can be completed online using https://www.goodreports.org/, a tool made by the EQUATOR Network in collaboration with Penelope.ai

For peer review only

Reporting checklist for randomised trial.

Based on the CONSORT guidelines.

Instructions to authors

Complete this checklist by entering the page numbers from your manuscript where readers will find each of the items listed below.

Your article may not currently address all the items on the checklist. Please modify your text to include the missing information. If you are certain that an item does not apply, please write "n/a" and provide a short explanation.

Upload your completed checklist as an extra file when you submit to a journal.

In your methods section, say that you used the CONSORTreporting guidelines, and cite them as:

Schulz KF, Altman DG, Moher D, for the CONSORT Group. CONSORT 2010 Statement: updated guidelines for reporting parallel group randomised trials

		Reporting Item	Page Number
**Title and Abstract**			
Title	#1a	Identification as a randomized trial in the title.	1
Abstract	#1b	Structured summary of trial design, methods, results, and conclusions	1
**Introduction**			
Background and objectives	#2a	Scientific background and explanation of rationale	2-3
Background and objectives	#2b	Specific objectives or hypothesis	3
**Methods**			
Trial design	#3a	Description of trial design (such as parallel, factorial) including allocation ratio.	4
Trial design	#3b	Important changes to methods after trial commencement (such as eligibility criteria), with reasons	n/a

For peer review only

Participants	#4a	Eligibility criteria for participants	5-6
Participants	#4b	Settings and locations where the data were collected	4
Interventions	#5	The experimental and control interventions for each group with sufficient details to allow replication, including how and when they were actually administered	7
Outcomes	#6a	Completely defined prespecified primary and secondary outcome measures, including how and when they were assessed	11
Sample size	#7a	How sample size was determined.	10
Sample size	#7b	When applicable, explanation of any interim analyses and stopping guidelines	10
Randomization - Sequence generation	#8a	Method used to generate the random allocation sequence.	
7			
Randomization - Sequence generation	#8b	Type of randomization; details of any restriction (such as blocking and block size)	
7			
Randomization - Allocation concealment mechanism	#9	Mechanism used to implement the random allocation sequence (such as sequentially numbered containers), describing any steps taken to conceal the sequence until interventions were assigned	7
Randomization - Implementation	#10	Who generated the allocation sequence, who enrolled participants, and who assigned participants to interventions	7
Blinding	#11a	If done, who was blinded after assignment to interventions (for example, participants, care providers, those assessing outcomes) and how.	8
Blinding	#11b	If relevant, description of the similarity of interventions	8
Statistical methods	#12a	Statistical methods used to compare groups for primary and secondary outcomes	11
Statistical methods	#12b	Methods for additional analyses, such as subgroup analyses and adjusted analyses	11

For peer review only

Outcomes	#6b	Any changes to trial outcomes after the trial commenced, with reasons	n/a
**Results**			
Participant flow diagram (strongly recommended)	#13a	For each group, the numbers of participants who were randomly assigned, received intended treatment, and were analysed for the primary outcome	n/a
Participant flow	#13b	For each group, losses and exclusions after randomization, together with reason	n/a
Recruitment	#14a	Dates defining the periods of recruitment and follow-up	n/a
Recruitment	#14b	Why the trial ended or was stopped	n/a
Baseline data	#15	A table showing baseline demographic and clinical characteristics for each group	n/a
Numbers analysed	#16	For each group, number of participants (denominator) included in each analysis and whether the analysis was by original assigned groups	n/a
Outcomes and estimation	#17a	For each primary and secondary outcome, results for each group, and the estimated effect size and its precision (such as 95% confidence interval)	n/a
Outcomes and estimation	#17b	For binary outcomes, presentation of both absolute and relative effect sizes is recommended	n/a
Ancillary analyses	#18	Results of any other analyses performed, including subgroup analyses and adjusted analyses, distinguishing pre-specified from exploratory	n/a
Harms	#19	All important harms or unintended effects in each group (For specific guidance see CONSORT for harms)	n/a
**Discussion**			
Limitations	#20	Trial limitations, addressing sources of potential bias, imprecision, and, if relevant, multiplicity of analyses	11
Interpretation	#22	Interpretation consistent with results, balancing benefits and harms, and considering other relevant evidence	n/a
Registration	#23	Registration number and name of trial registry	12

For peer review only

Generalisability	#21	Generalisability (external validity, applicability) of the trial findings	n/a

Other information

Interpretation	#22	Interpretation consistent with results, balancing benefits and harms, and considering other relevant evidence	n/a
Registration	#23	Registration number and name of trial registry	12
Protocol	#24	Where the full trial protocol can be accessed, if available	12
Funding	#25	Sources of funding and other support (such as supply of drugs), role of funders	13

The CONSORT checklist is distributed under the terms of the Creative Commons Attribution License CC-BY. This checklist was completed on 10. August 2023 using https://www.goodreports.org/, a tool made by the EQUATOR Network in collaboration with Penelope.ai

For peer review only
